# Bone Safety During the First Ten Years of Gender‐Affirming Hormonal Treatment in Transwomen and Transmen

**DOI:** 10.1002/jbmr.3612

**Published:** 2018-12-07

**Authors:** Chantal M Wiepjes, Renate T de Jongh, Christel JM de Blok, Mariska C Vlot, Paul Lips, Jos WR Twisk, Martin den Heijer

**Affiliations:** ^1^ Department of Internal Medicine VU University Medical Center Amsterdam the Netherlands; ^2^ Center of Expertise on Gender Dysphoria VU University Medical Center Amsterdam the Netherlands; ^3^ Department of Clinical Epidemiology VU University Amsterdam the Netherlands

**Keywords:** TRANSGENDER, BONE, OSTEOPOROSIS, GENDER‐AFFIRMING HORMONAL TREATMENT, DXA

## Abstract

Concerns about the effects of gender‐affirming hormonal treatment (HT) on bone mineral density (BMD) in transgender people exist, particularly regarding the decrease in estrogen concentrations in transmen. Although it is known that HT is safe for BMD in the short term, long‐term follow‐up studies are lacking. Therefore this study aimed to investigate the change in BMD during the first 10 years of HT, to determine whether HT is safe and if assessing BMD during HT is necessary. A follow‐up study was performed in adult transgender people receiving HT at the VU University Medical Center Amsterdam between 1998 and 2016. People were included if they were HT naive and had a dual‐energy X‐ray absorptiometry (DXA) scan at the start of HT. Follow‐up DXA scans performed after 2, 5, and/or 10 years of HT were used for analyses. The course of BMD of the lumbar spine during the first 10 years of HT was analyzed using multilevel analyses. A total of 711 transwomen (median age 35 years; IQR, 26 to 46 years) and 543 transmen (median age 25 years; IQR, 21 to 34 years) were included. Prior to the start of HT, 21.9% of transwomen and 4.3% of transmen had low BMD for age (*Z*‐score < –2.0). In transwomen lumbar spine BMD did not change (+0.006; 95% CI, –0.005 to +0.017), but lumbar spine *Z*‐score increased by +0.22 (95% CI, +0.12 to +0.32) after 10 years of HT. Also in transmen lumbar spine BMD did not change (+0.008; 95% CI, –0.004 to +0.019), but lumbar spine *Z*‐score increased by +0.34 (95% CI, +0.23 to +0.45) after 10 years of HT. This study showed that HT does not have negative effects on BMD, indicating that regularly assessing BMD during HT is not necessary. However, a high percentage of low BMD was found prior to HT, especially in transwomen. Therefore, evaluation of BMD before start of HT may be considered. © 2018 The Authors. *Journal of Bone and Mineral Research* Published by Wiley Periodicals, Inc.

## Introduction

Sex hormones influence bone acquisition and metabolism. Men develop wider bones and greater cortical bone size than women due to periosteal apposition.^1^, ^2^ In women, the decline in estrogen during menopause leads to an increase in bone resorption^3^ and a decrease in bone mineral density (BMD).^4^ A higher trabecular BMD was found in women with androgen excess,^5^ indicating that testosterone also influences BMD in women. In men, it was found that testosterone deficiency following orchiectomy was associated with accelerated bone loss.^6^ However, more recent studies indicated that the effects of hypogonadism on bone in men are mainly due to estrogen instead of testosterone deficiency.^7^, ^8^ In both hypogonadal men and women, treatment with sex hormones increases BMD.^9^, ^10^

Gender‐affirming hormonal treatment (HT) in transgender people influences bone metabolism. After 1 year of HT the BMD increases in transwomen (male‐to‐female transgender people),^11^, ^12^, ^13^, ^14^, ^15^, ^16^, ^17^, ^18^, ^19^ whereas in transmen (female‐to‐male transgender people) a maintenance^12^, ^17^, ^19^, ^20^, ^21^ or increase^16^ in BMD is described. More specifically, a larger increase in BMD was found in postmenopausal transmen with estrogen deficiency prior to HT compared with premenopausal transmen with normal estradiol concentrations. This suggests that the increase in BMD in transmen is mainly caused by the aromatization of testosterone into estradiol, therefore increasing the estradiol concentrations, instead of the direct effects of testosterone.^16^

The long‐term effects of HT on BMD have been investigated using small‐sample (*n* < 50) cross‐sectional case‐control studies, with contradictory results. In transwomen compared with control men, higher,^22^ similar,^23^ and lower^24^ BMD was found after 17, 12, and 8 years of HT, respectively. Also, in transmen compared with control women contradictory results are published: one study found no difference in BMD after 10 years of HT,^25^ whereas another study found higher whole‐body *Z*‐scores after 7.6 years of HT.^23^ Even prior to HT differences are observed: transwomen had lower BMD compared with control men,^12^, ^26^ whereas transmen had higher^12^ or similar^25^ BMD than control women, indicating that the difference in BMD after long‐term HT might already be present at baseline.

The transgender population who seek help is steeply growing,^27^ which leads to an increasing demand for knowledge about the safety of HT, including bone health. Although multiple studies investigated BMD after a longer period of HT, no studies have investigated the course of BMD during long‐term HT. Therefore, our study aimed to describe BMD prior to the start of HT in a large population of transwomen and transmen, to investigate if BMD changes during the first 10 years of HT, and as a result, to generate information about whether it is necessary to assess bone health in trans people during regular patient care. Furthermore, we studied whether different age groups and sex hormone concentrations were associated with a change in BMD during HT.

## Patients and Methods

### Design and population

**Figure 1:**
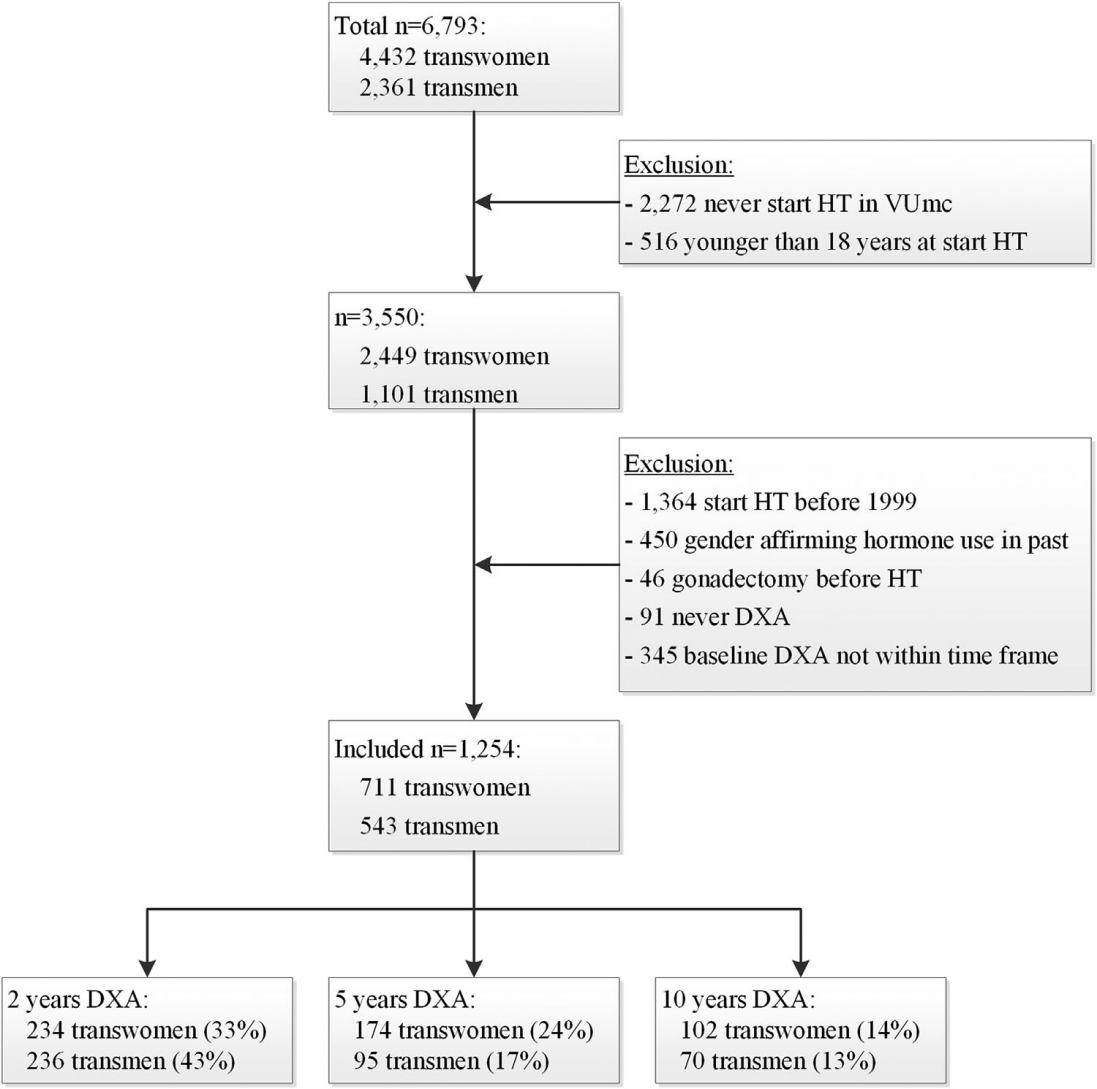
Flowchart of inclusion of study population. DXA not within time frame: more than 1 year before start of gender‐affirming hormonal treatment or more than 4 months after start of HT. The reported percentages are the percentages of transwomen or transmen with a DXA scan at that time point compared with the total included transwomen or transmen, respectively. DXA = dual‐energy X‐ray absorptiometry; HT = gender‐affirming hormonal treatment; VUmc = VU University medical center.

This study is part of the retrospective Amsterdam Cohort of Gender Dysphoria (ACOG) study, including all 6793 people who once presented themselves at the gender identity clinic of the VU University Medical Center (VUmc), Amsterdam, the Netherlands, between 1972 and 2016. Study design has been described.^27^ Briefly, data was collected by reviewing medical records. Age, medical history, medication use, type and dose of HT, prior HT use, gender‐affirming surgery, smoking habits (in cigarettes per day), body weight (in kilograms), body height (in centimeters), laboratory test results, and dual‐energy X‐ray absorptiometry (DXA) results were retrieved. HT consisted of oral or transdermal estrogens and, usually until gonadectomy, anti‐androgens in transwomen. Transmen were treated with oral, transdermal, or intramuscular testosterone. After at least 1 to 1.5 years of HT, surgery could be performed. For the current study, only people 18 years and older with a baseline DXA scan (range 1 year before to 4 months after start of HT) who started with HT after 1998 were included, because BMD measurements were available since then. People who had undergone gonadectomy (orchiectomy in transwomen or oophorectomy in transmen) or used gender‐affirming hormones prior to HT were excluded. We calculated that with 711 included transwomen and 543 included transmen (Fig. 1), a mean difference in lumbar spine BMD of 0.021 g/cm^2^ and 0.022 g/cm^2^ could be detected, respectively, with a power of 80% and an alpha of 0.05. Our local ethics committee reviewed the study and determined that the Medical Research Involving Human Subjects Act (WMO) did not apply to this study, and necessity for informed consent was waived due to the retrospective design.

### BMD

BMD measurements were performed during regular patient care before the start of HT and were usually repeated every 2 to 5 years during HT. BMD of the lumbar spine (LS), total hip (TH), and femoral neck (FN) were measured using a DXA Hologic Delphi, which was updated in July 2004 and replaced by a Hologic Discovery A in February 2011 (Hologic Inc., Bedford, MA, USA). Phantom calibration allowed for comparison of BMD values with a difference of <1.0% between the densitometers. Coefficients of variation were <1.0% for both densitometers and least significant change was 0.022 g/cm^2^. Absolute BMD values (g/cm^2^) were obtained and were converted into *T*‐scores (standard deviation [SD] difference between the person's BMD and the BMD of a young‐adult reference population of the same birth‐sex and ethnicity) and *Z*‐scores (SD difference between the person's BMD and an age‐matched BMD of the same birth‐sex and ethnicity), based on the National Health and Nutrition Examination Survey (NHANES). In addition, *T*‐scores and *Z*‐scores on baseline were also calculated using the identified sex as reference population. Osteoporosis was defined as a *T*‐score below –2.5, and a *Z*‐score below –2.0 was classified as low bone density for age.^28^

### Biochemical assessments

Blood samples were obtained during regular patient care before the start of HT, after 3 months of HT, and thereafter usually every 1 to 2 years. Higher‐quality assays were implemented when available, and conversion formulas were generated to allow for comparison of the old and new values.

Estradiol was measured using a radioimmunoassay (Diasorin, Saluggia, Italy) with a lower limit of quantitation (LOQ) of 18 pmol/L and an interassay coefficient of variation (CV) of 10% until January 2010, after which a competitive immunoassay (Delfia; Wallac, Turku, Finland) was used until July 2014 (LOQ 20 pmol/L, interassay CV 10%). The formula Delfia = 1.267 × Diasorin–28.87 was used for conversion. After July 2014, LC‐MS/MS (VUmc, Amsterdam, the Netherlands) was used (LOQ 20 pmol/L, interassay CV 7%). For conversion, the formula LC‐MS/MS = 1.60 × Delfia–29 was used.

Testosterone was measured using a radioimmunoassay (RIA; Coat‐A‐Count; Siemens Medical Solutions USA, Inc., Malvern, PA, USA) until January 2013 (LOQ 1 nmol/L, interassay CV 7% to 20%). Thereafter, a competitive immunoassay (Architect; Abbott, Abbott Park, IL, USA) was used (LOQ 0.1 nmol/L, interassay CV 6% to 10%). For conversion, two formulas were used: Architect = 1.1 × RIA+0.2 for testosterone concentrations <8 nmol/L; Architect = 1.34 × RIA–1.65 for testosterone concentrations >8 nmol/L.

Luteinizing hormone (LH) was measured using an immunometric assay (Delfia; Wallac, Turku, Finland) until June 2011 (LOQ 0.5 U/L, interassay CV <7%). Thereafter, an immunometric assay (Architect; Abbott, Abbott Park, IL, USA) was used (LOQ 2 U/L, interassay CV <4% to 6%). For conversion, the formula Architect = 0.91 × Delfia–0.01 was used.

### Statistical analyses

Baseline characteristics are reported as mean ± SD, median (interquartile range [IQR]), or percentages. Independent *t* tests (or Wilcoxon rank sum tests in case of non‐normal distribution) or chi‐square tests were used to analyze the differences in baseline characteristics between people with at least one follow‐up measurement and people without a follow‐up measurement.

Linear multilevel analyses with measurements clustered within people were performed to analyze the course of absolute BMD values and *Z*‐scores during the first 10 years of HT. Time was analyzed as a categorical variable with set time points at baseline (range, 1 year before to 4 months after baseline), after 2 years of HT (range, after 1 to 3 years of HT), after 5 years of HT (range, after 3 to 7.5 years of HT), or after 10 years of HT (range, after 7.5 to 12 years of HT) using dummy variables. Next, stratified analyses were performed to investigate the change in BMD over time for different age groups (<30 years, 30 to 40 years, and ≥40 years), sex hormone concentrations during HT, and baseline BMD. The mean estradiol, testosterone, and LH concentrations during HT were calculated by averaging the results from the laboratory measurements after 1, 2, 5, and 10 years of HT (number of measurements, mean ± SD, 2.5 ± 0.8). Because no linear associations existed between change in BMD and hormone concentrations, estradiol concentrations were divided into tertiles, LH concentrations into suppressed (<1 U/L) or not suppressed (>1 U/L), and testosterone concentrations into suppressed (<2 nmol/L) or not suppressed (>2 nmol/L) for transwomen, and tertiles for transmen. Baseline BMD was divided into tertiles. Last, the multilevel analyses were repeated by adding age, estradiol, testosterone, and LH concentrations as interactions with time to the model.

For all analyses, STATA Statistical Software (StataCorp, College Station, TX, USA) version 13.1 was used.

## Results

### Study participants

The flowchart of the inclusion of participants is shown in Fig. 1. In total, 711 transwomen and 543 transmen with a baseline LS DXA were included for analyses, of which 440 transwomen and 358 transmen also had at least one follow‐up DXA. No differences were found in age, ethnicity, BMD and *Z*‐score of the LS, estradiol, testosterone, LH, and 25 (OH)D concentrations at baseline between people with and without follow‐up BMD measurements (data not shown). The baseline characteristics of the study population are shown in Table 1.

**Table 1:** Baseline Characteristics of the Study Population

Baseline characteristics	Transwomen		Transmen	
Age (years), median (IQR)	35 (26–46)		25 (21–34)	
Ethnicity, white (%)	97.2		95.2	
BMI (kg/m^2^), mean ± SD	23.7 (4.3)		25.6 (5.7)	
Smoking, yes (%)	34.9		39.5	
**Bone mineral density**	Male reference	Female reference	Male reference	Female reference
Lumbar spine, mean ± SD				
Absolute BMD (g/cm^2^)	0.976 ± 0.140		1.030 ± 0.127	
*T*‐score	–1.07 ± 1.27	–0.67 ± 1.27	–0.61 ± 1.14	–0.20 ± 1.14
*Z*‐score	–0.93 ± 1.32	–0.31 ± 1.39	–0.54 ± 1.15	+0.01 ± 1.14
Total hip, mean ± SD				
Absolute BMD (g/cm^2^)	0.928 ± 0.136		0.948 ± 0.118	
*T*‐score	–0.72 ± 0.89	–0.13 ± 1.09	–0.61 ± 0.75	+0.01 ± 0.92
*Z*‐score	–0.58 ± 0.92	+0.07 ± 1.16	–0.55 ± 0.76	+0.07 ± 0.93
Femoral neck, mean ± SD				
Absolute BMD (g/cm^2^)	0.789 ± 0.129		0.838 ± 0.118	
*T*‐score	–1.06 ± 0.93	–0.56 ± 1.14	–0.72 ± 0.83	–0.14 ± 1.02
*Z*‐score	–0.73 ± 0.94	–0.25 ± 1.16	–0.59 ± 0.85	–0.05 ± 1.02
Osteoporosis (%)	14.2	5.8	5.2	2.4
Low bone density (%)	21.9	9.4	10.3	4.3
**Laboratory measurements** ^a^	Baseline	During HT	Baseline	During HT
Estradiol (pmol/L), median (IQR)	95 (68–124)	235 (160–338)	185 (59–390)	159 (113–220)
Testosterone (nmol/L), median (IQR)	20 (16–25)	1.1 (0.7–1.3)	1.3 (1.2–1.7)	26 (18–38)
LH (U/L), median (IQR)	3.4 (2.3–4.6)	1.2 (0.1–6.2)	4.2 (2.4–7.1)	3.3 (0.7–8.8)
25(OH)D (nmol/L), median (IQR)	42 (26–58)	53 (35–72)	50 (30–73)	57 (40–78)
Calcium (mmol/L), mean ± SD	2.36 (0.08)	2.32 (0.08)	2.34 (0.08)	2.36 (0.08)
Creatinine (µmol/L), mean ±** **SD	76 (11)	72 (11)	66 (10)	78 (11)
AF (U/L), mean ± SD	71 (19)	67 (23)	70 (22)	78 (21)
SHBG (nmol/L), median (IQR)	35 (26–46)	43 (29–59)	51 (31–81)	28 (20–36)

Values are median (IQR), mean ± SD, or percentage.

IQR = interquartile range; BMD = bone mineral density; LH = luteinizing hormone; 25(OH)D = 25‐hydroxy vitamin D; AF = alkaline phosphatase; SHBG = sex‐hormone binding globulin.

^a^ Concentrations were not known for the entire population. Available percentage at baseline: estradiol, testosterone, and LH (75% to 85%); 25(OH)D, calcium, and creatinine (45% to 70%); and AF and SHBG (15% to 25%). During HT: estradiol, testosterone, LH, creatinine (>90%); calcium and AF (75% to 90%); and 25(OH)D and SHBG (50% to 60%).

### Lumbar spine

#### Transwomen on estradiol‐based treatment

Transwomen had a mean LS *Z*‐score of −0.93 ± 1.32 before start of HT. At baseline, 14.2% of the transwomen were classified as having osteoporosis (*T*‐score ≤ –2.5), and 21.9% had low bone density (*Z*‐score < –2.0) for age.

**Figure 2:**
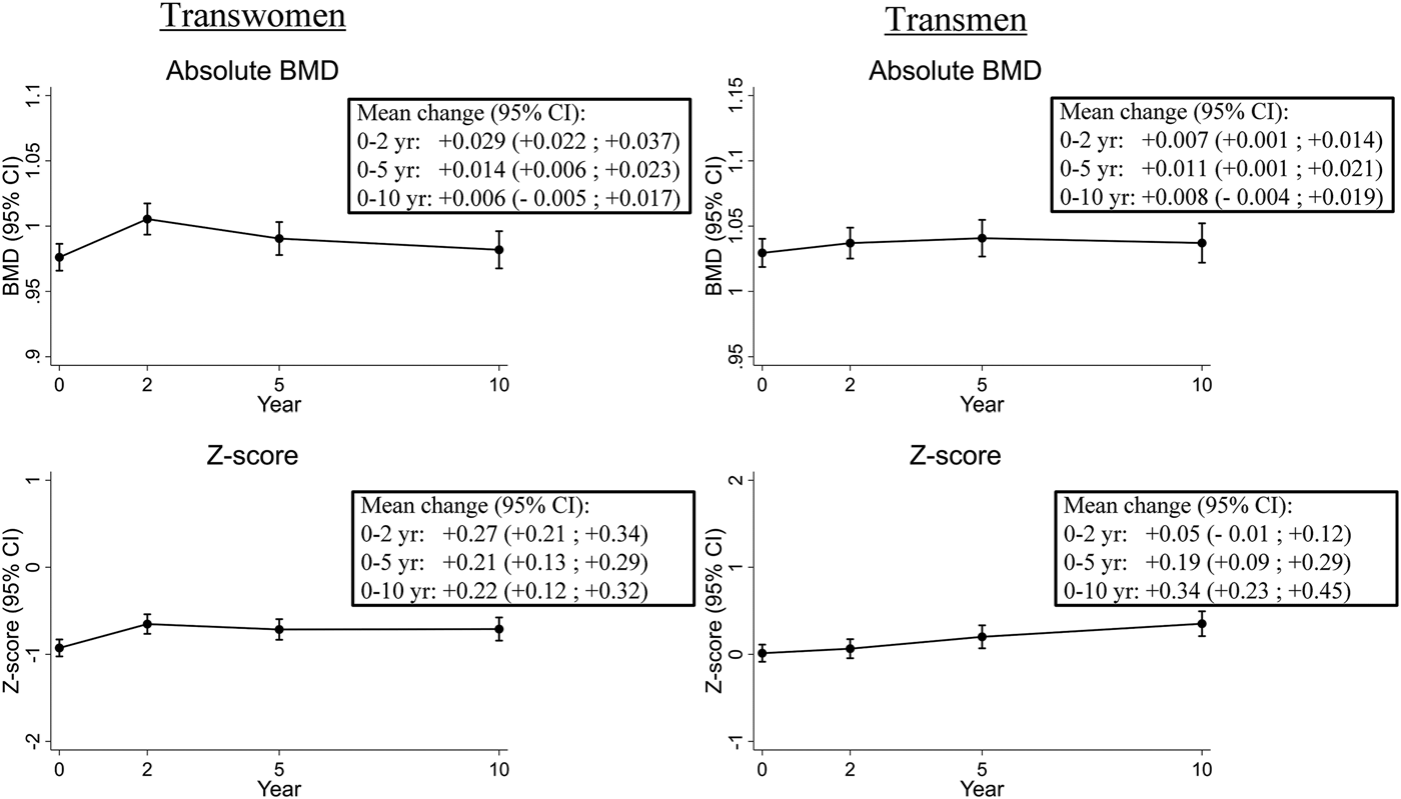
Change in absolute BMD and *Z*‐score in transwomen and transmen during the first 10 years of gender‐affirming hormonal treatment. CI = confidence interval; BMD = bone mineral density; yr = year.

**Figure 3:**
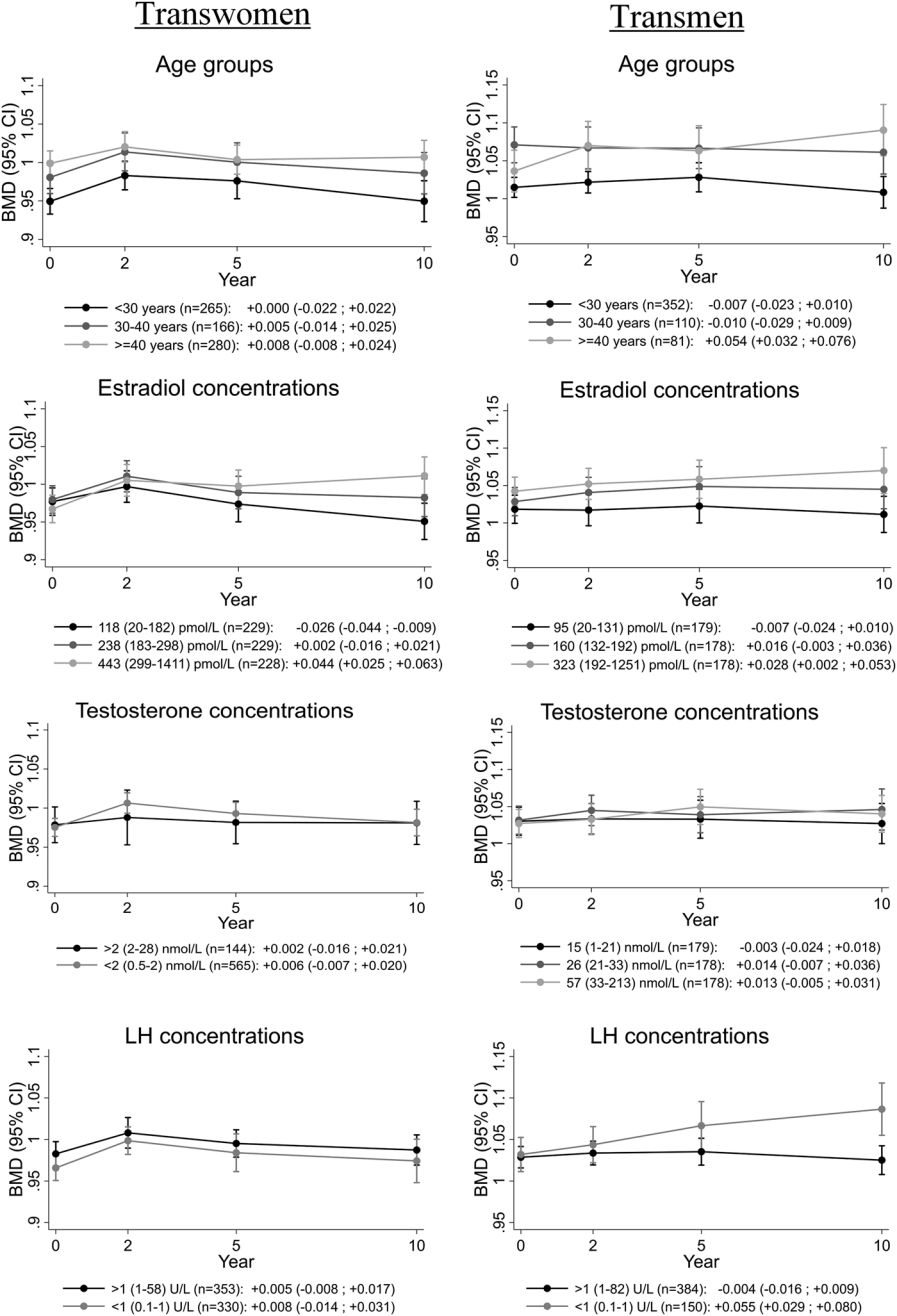
Change in BMD during the first 10 years of gender‐affirming hormonal treatment in transwomen and transmen, stratified for age groups, concentrations of estradiol, testosterone, and LH. For each group, the mean change with 95% CI is reported below the graphs. Age is defined as age at start of HT. Estradiol, testosterone, and LH concentrations were assessed during HT and are shown as mean (range). BMD = bone mineral density; CI = confidence interval; LH = luteinizing hormone.

After 10 years of HT, LS BMD was not different from baseline (+0.006 g/cm^2^; 95% CI, –0.005 to +0.017 g/cm^2^), but LS *Z*‐score was +0.22 (95% CI, +0.12 to +0.32) higher compared with baseline (Fig. 2). As shown in Fig. 3, no differences in change in LS BMD were observed between different age groups. In transwomen in the highest estradiol tertile (mean 443 pmol/L), an increase in LS BMD was observed (+0.044 g/cm^2^; 95% CI, +0.025 to +0.063 g/cm^2^), whereas it decreased in those in the lowest tertile (mean 118 pmol/L: –0.026 g/cm^2^; 95% CI, –0.044 to –0.009 g/cm^2^). There was no difference in change in LS BMD between suppressed versus not suppressed testosterone and LH. Transwomen in the lowest baseline BMD tertile (mean, 0.828 g/cm^2^) increased by +0.026 (95% CI, +0.004 to +0.048), whereas no change was observed in those in the middle (mean 0.972 g/cm^2^: change –0.014; 95% CI, –0.031 to +0.003) or highest tertile (mean 1.129 g/cm^2^: change +0.006; 95% CI, –0.010 to +0.022).

In the multivariable analysis (Table 2), no differences in change in LS BMD were found between age groups, testosterone concentrations, and LH concentrations. Higher estradiol tertiles were associated with a larger increase in LS BMD than lower estradiol tertiles.

**Table 2:** Adjusted Differences of the Change in Lumbar Spine Bone Mineral Density Between Baseline and 10 Years

	Transwomen Difference in change (95% CI) (g/cm^2^)	Transmen Difference in change (95% CI) (g/cm^2^)
Age		
30–40 years versus <30 years	+0.008 (–0.020 to +0.037)	–0.007 (–0.033 to +0.018)
>40 years versus <30 years	–0.004 (–0.031 to +0.024)	+0.052 (+0.023 to +0.081)
>40 years versus 30–40 years	–0.012 (–0.036 to +0.013)	+0.059 (+0.030 to +0.088)
Estradiol concentration		
Second tertile versus first tertile	+0.033 (+0.006 to +0.059)	+0.009 (–0.017 to +0.034)
Third tertile versus first tertile	+0.076 (+0.050 to +0.103)	+0.025 (–0.005 to +0.055)
Third tertile versus second tertile	+0.044 (+0.018 to +0.070)	+0.017 (–0.014 to +0.047)
LH concentration		
Suppressed versus not suppressed	–0.008 (–0.033 to +0.017)	+0.043 (+0.013 to +0.073)
Testosterone concentration		
Suppressed versus not suppressed	–0.011 (–0.034 to +0.012)	
Second tertile versus first tertile	–	+0.013 (–0.016 to +0.041)
Third tertile versus first tertile	–	–0.002 (–0.030 to +0.025)
Third tertile versus second tertile	–	–0.015 (–0.043 to +0.012)

Age is defined as age at start of HT. Number of transwomen per group: <30 years (*n* = 265); 30–40 years (*n* = 166); ≥40 years (*n* = 280). Number of transmen per group: <30 years (*n* = 352); 30–40 years (*n* = 110); ≥40 years (*n* = 81). Estradiol, testosterone, and LH concentrations were assessed during HT. In transwomen, the mean (range) estradiol concentrations were 118 (20–182) pmol/L for the 1st tertile (*n* = 229); 238 (182–298) pmol/L for the 2nd tertile (*n* = 229); and 443 (229–1411) pmol/L for the 3rd tertile (*n* = 228). Testosterone concentration was divided into suppressed (<2 nmol/L, *n* = 565) and not suppressed (>2 nmol/L, *n* = 144), and LH concentration was divided into suppressed (<1 U/L, *n* = 330) and not suppressed (>1 U/L, *n* = 353). In transmen, the mean (range) estradiol concentrations were 95 (20–131) pmol/L for the 1st tertile (*n* = 179); 160 (132–192) pmol/L for the 2nd tertile (*n* = 178); and 323 (192–1251) pmol/L for the 3rd tertile (*n* = 178). The mean (range) testosterone concentrations were 15 (1–21) nmol/L for the 1st tertile (*n* = 179); 26 (21–33) nmol/L for the 2nd tertile (*n* = 178); and 57 (33–213) nmol/L for the 3rd tertile (*n* = 178). LH concentration was divided into suppressed (<1 U/L, *n* = 150) or not suppressed (>1 U/L, *n* = 384).

CI = confidence interval.

#### Transmen on testosterone‐based treatment

The baseline LS *Z*‐score was +0.01 ± 1.14. Osteoporosis (*T*‐score ≤ –2.5) was found in 2.4%, and low bone density for age (*Z*‐score < –2.0) in 4.3% at baseline.

LS BMD after 10 years of HT was similar to baseline (+0.008 g/cm^2^; 95% CI, –0.004 to +0.019 g/cm^2^), but LS *Z*‐score increased by +0.34 (95% CI, +0.23 to +0.45) from baseline to 10 years (Fig. 2). In the oldest age group (≥40 years) LS BMD increased (+0.054 g/cm^2^; 95% CI, +0.032 to +0.076 g/cm^2^), whereas it did not change in the younger two age groups. Baseline estradiol concentrations were lower in transmen ≥40 years (median 104 pmol/L; IQR, 20 to 386 pmol/L) than younger transmen (median 197 pmol/L; IQR, 77 to 390 pmol/L). No change in LS BMD was found in transmen who were categorized in the two lowest estradiol tertiles (means 95 and 160 pmol/L, respectively), but it increased in those who were categorized in the highest estradiol tertile (mean 323 pmol/L, Fig. 3). Testosterone concentrations were not associated with change in LS BMD. An increase in LS BMD was found in transmen with suppressed LH concentrations (<1 U/L), whereas no change was observed in those with higher LH concentrations (Fig. 3). Transmen in the lowest baseline BMD tertile (mean 0.896 g/cm^2^) increased in BMD (+0.030; 95% CI, +0.010 to +0.051), whereas it did not change in the middle tertile (mean 1.021 g/cm^2^: change +0.004; 95% CI, –0.018 to +0.026) or highest tertile (mean 1.172 g/cm^2^: change –0.005; 95% CI, –0.020 to +0.010).

In the multivariable analysis (Table 2), estradiol concentrations were no longer associated with the change in LS BMD. Testosterone was not associated with change in LS BMD. Older transmen had a larger increase in LS BMD compared with younger transmen. Transmen with lower LH concentrations had a larger increase in LS BMD compared to those with higher LH concentrations.

### TH and FN

Only a small number of BMD measurements of TH and FN were performed after 5 years (*n* = 24) and 10 years (*n* = 4) of HT. In the overall analyses, no changes in TH BMD (–0.034 g/cm^2^; 95% CI, –0.070 to +0.002 g/cm^2^) and FN BMD (+0.057 g/cm^2^; 95% CI, –0.036 to +0.047 g/cm^2^) were found in transwomen. Also in transmen, no changes in TH BMD (–0.024 g/cm^2^; 95% CI, –0.075 to +0.026 g/cm^2^) and FN BMD (–0.033 g/cm^2^; 95% CI, –0.092; +0.026 g/cm^2^) were found. No subgroup analyses could be performed due to the limited number of people in each of the subgroups.

## Discussion

In this study, we found that transwomen had a low mean LS *Z*‐score before the start of HT, whereas this was not found in transmen. During the first 10 years of HT, no change in LS BMD was found, whereas the LS *Z*‐score increased in both transwomen and transmen. Higher estradiol concentrations were associated with an increase in LS BMD in transwomen, whereas in transmen lower LH concentrations were associated with an increase in LS BMD.

A low bone density in transwomen before the start of HT was earlier described by van Caenegem and colleagues,^26^ who found that transwomen had lower 25(OH)D concentrations and lower muscle mass than control men, possibly due to less activities because of social isolation. Although we did not have a control group, we did find that transwomen had lower 25(OH)D concentrations at baseline than transmen.

Because BMD is influenced by time, it was also analyzed as *Z*‐score in order to compare the results with the general age‐matched population. For the current study, the *Z*‐score was calculated using the BMD of the sex assigned at birth, because the majority of the population started hormonal treatment after puberty and therefore had already reached the age of peak bone mass that belongs to the sex assigned at birth. The natural course of BMD over time is to decrease after the peak bone mass, whereas we found that LS BMD did not change during the first 10 years of HT. In addition, the LS *Z*‐score increased, which may indicate that HT does not negatively influence BMD.

In transwomen, no differences in change in LS BMD between age groups were present, whereas in transmen LS BMD increased more in the oldest age group (≥40 years) compared with younger age groups. This finding was also described in a prospective 1‐year follow‐up study.^16^ In the current study, we found that the increase continued after 1 year. An explanation for the larger increase in LS BMD in this age group may be that these people were perimenopausal or postmenopausal with low estrogen concentrations prior to HT. Treatment with testosterone increased both testosterone and estradiol concentrations in these transmen (due to aromatization of testosterone to estradiol), whereas in the younger transmen only testosterone concentrations increased as they were not estrogen deficient at baseline. This may indicate that the effects of HT on BMD in transmen were mainly due to indirect effects of estradiol instead of direct effects of testosterone. This is also in line with earlier studies who reported that BMD was better correlated with estradiol concentrations than testosterone concentrations.^7^, ^29^ Moreover, when we adjusted this analysis for change in estradiol concentrations (mean estradiol concentration during HT minus estradiol concentration at baseline), no differences between age groups remained present (data not shown). This indicates that the difference in change in BMD between age groups is (partially) explained by a difference in change in estradiol concentrations.

In transwomen with higher estradiol concentrations during HT an increase in LS BMD was found, whereas low estradiol concentrations were associated with a decrease in LS BMD. Testosterone concentrations were suppressed in the majority of the population and were not associated with the change in LS BMD. LH concentrations were not associated with change in LS BMD, but were also suppressed by the use of cyproterone acetate and is therefore not indicative of adequate estrogen substitution. In transmen, estradiol and testosterone concentrations were not associated with change in LS BMD. In transmen with low LH concentrations the LS BMD increased, whereas it did not change in those with higher LH concentrations. These results indicate that in transmen, LH concentrations may be used to evaluate the adequacy of testosterone dosing for bone health, whereas in transwomen estradiol concentrations may be used to evaluate the adequacy of estradiol dosing. In addition, because LS BMD decreased in transwomen with low estradiol concentrations, therapy compliance should be stimulated.

Our study was performed in the largest gender identity clinic of the Netherlands, including a large population of transwomen and transmen with a wide age range. Previous studies investigated the long‐term effects of HT on BMD in cross‐sectional studies, without baseline differences taken into account. In this study, long‐term follow‐up analyses were performed, also looking at the influence of age and sex hormone concentrations. However, there are also some limitations. First, because this study is a retrospective study, data were collected during regular patient care. Clinical data, such as smoking habits, alcohol intake, and body weight, were usually only assessed at baseline but not structurally during medical checkups and could not be reliably analyzed. Also, data about calcium intake, vitamin D supplementation, or physical activity were not available, which could also influence BMD. It might be possible that trans people gain a healthier lifestyle during HT, for example more exercising, quit smoking, and using vitamin D supplementation. Because a control group was lacking, the change in BMD cannot only be contributed to the use of HT. Although this study is retrospective, LS BMD measurements and sex hormone concentrations were regularly assessed as this was part of the treatment protocol. For this study, people were included for analyses if they had a DXA scan prior to the start of HT. No differences in baseline characteristics were observed between people with and without follow‐up LS BMD measurements, which decreases the risk of selection bias. Second, differences in change in BMD for different treatment regimens were not analyzed. Because trans people often change the type of HT they use, it could not be analyzed whether the change in BMD differs for people who use transdermal, oral, or intramuscular estrogen or testosterone. In addition, the majority of the population underwent gonadectomy during the first 10 years of HT,^27^ so the influence of surgery on change in BMD could not be analyzed. Third, in this study only the LS BMD was analyzed in subgroup analyses, because not enough data were available for subgroup analyses of the TH or FN BMD. However, the overall analyses showed the same pattern as the LS analysis, so it is not expected that it would give different conclusions. Fourth, the type of densitometer changed during the study period, whereas most ideally all people would be scanned by the same densitometer. However, during replacement of the densitometer, phantom calibration was performed and a difference of less than 1% in absolute BMD values between the densitometers was found. This indicates that the change in densitometer did not affect our result. Last, only the change in BMD was assessed and no fracture data was available. Because HT does not seem to have negative effects on BMD, it is not expected that fracture risk will increase because of HT.

In clinical practice, concerns about the safety of HT on bone health are present, probably because earlier studies found low BMD after long‐term HT in transwomen compared with control men, and because of fear that HT decreases estradiol concentrations in transmen, which could have negative effects on BMD. The current results may indicate that HT does not have negative effects on bone health in trans people, because no change in LS BMD and an increase in LS *Z*‐score was found. However, because prior to HT a high percentage of low bone density was found in transwomen, bone health should be an important topic, especially if therapy noncompliance is suspected. The results of this study support the statements of the Endocrine Society Guideline^30^ that clinicians should assess BMD only when risk factors for osteoporosis exists, and especially in those who stop HT after gonadectomy. For transwomen, testing BMD at baseline should be considered based on the high prevalence of low bone density at baseline, whereas this does not seem necessary for transmen. Regularly assessing BMD during HT without indication does not seem necessary based on the current results. However, the effect of HT on fracture risk is not known and is topic for further research.

## Disclosures

The authors have no relevant disclosures to declare.
